# Untreated Superior Vena Cava Aneurysm: Radiological Significance and Review of the Literature

**DOI:** 10.1155/2016/6960757

**Published:** 2016-12-05

**Authors:** Abhinav Patel, Ryan Cobb, Victor Rivera, Scott Simpson

**Affiliations:** ^1^Lewis Katz School of Medicine at Temple University, Philadelphia, PA, USA; ^2^Temple University Hospital Department of Diagnostic and Interventional Radiology, Philadelphia, PA, USA

## Abstract

Superior vena cava (SVC) aneurysms are a rare entity. The majority of the literature is in the form of case reports. SVC aneurysms are often an incidental finding with iatrogenic, congenital, or idiopathic etiologies. Treatment goals focus on preventing theoretical rupture or thrombus formation. Management options include observation, conservative medical management, surgical excision, and thrombin injection. We present a 73-year-old female with an incidental SVC aneurysm discovered on computed tomography (CT) of the thorax. The patient was observed without intervention for greater than 6 years. No complications were attributable to the SVC aneurysm during follow-up or over the course of the patient's life.

## 1. Introduction

Aneurysms of the SVC are rare with approximately 36 cases reported at the time of this literature review [1]. The earliest cases were diagnosed via diagnostic thoracotomy or venography after chest radiograph revealed a mediastinal abnormality [2]. More recent cases were incidentally diagnosed on cross-sectional imaging in patients without symptoms attributable to the aneurysm [2–4]. Patients with symptoms referable to an SVC aneurysm had clear iatrogenic causes [5], complications secondary to local mass effect [1], or pulmonary symptoms secondary to thromboembolism [4]. Treatment options focus on preventing or palliating the aforementioned complications with surgical excision and anticoagulation representing the mainstays of current management.

## 2. Case Presentation

**Figure 1:**
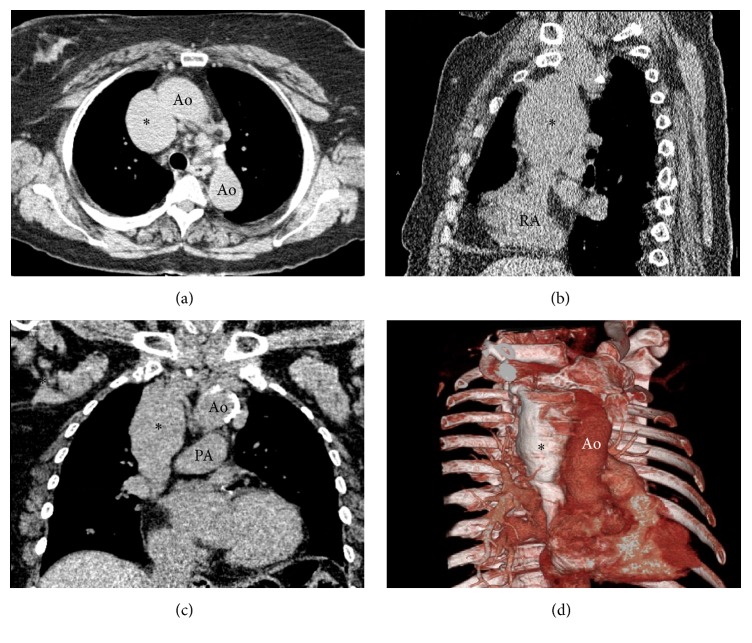
Axial (a), sagittal (b), and coronal (c) noncontrast CT images of the thorax demonstrating the SVC aneurysm (*∗*). (d) 3D reconstruction of the SVC aneurysm using CT of the thorax with IV contrast performed in 2014. Ao: aorta; PA: pulmonary artery; RA: right atrium.

A 73-year-old female presented as an outpatient from her primary care physician for a CT of the thorax, abdomen, and pelvis to evaluate a reportedly stable abdominal aortic aneurysm. Pertinent past medical history revealed a left supraclinoid internal carotid artery aneurysm, a stable infrarenal abdominal aortic aneurysm, systemic lupus erythematous/rheumatoid arthritis overlap syndrome, smoldering IgG lambda myeloma, chronic kidney disease (stage II), hyperparathyroidism, and a cerebral infarction in the right posterior cerebral artery territory in 2009. Findings on the CT of the thorax demonstrated a fusiform superior vena cava (SVC) aneurysm measuring 5.9 × 4.4 × 6.1 cm (anterior-posterior × transverse × craniocaudal). No thrombus was noted within the SVC aneurysm (Figure 1). The aneurysm was first partially visualized in 2007 on a cervical spine CT, and since that time it had remained stable on annual follow-up CTs.

The patient's course between 2007 and the most recent study in 2016 included an extensive rheumatologic and hematologic workup, including a temporal artery biopsy in 2015. Pathology revealed intimal hyperplasia with focal internal membrane disruption, but no features of granulomatous vasculitis were identified.

The etiology of the SVC aneurysm in this patient is uncertain. Potential causes in this patient include autoimmune disease (RA/SLE), which seems unlikely. Pathology did not reveal any convincing evidence of vasculitis. The arterial aneurysms are also felt to be unrelated as the patient has significant atherosclerotic disease. While an iatrogenic cause is possible, a chart review did not elicit any complications relating to prior hemodialysis catheter placement. Therefore, we surmised that the SVC aneurysm was likely idiopathic.

This patient did not receive any anticoagulation to prevent thrombus formation within the SVC aneurysm, and no repair was ever attempted. For the six years during which the patient was under observation at our institution, an SVC thrombus or sequela of an embolus originating from the SVC has never been observed. While the patient had a cerebral infarct in 2009, there was no evidence of right to left circulatory shunt. The observatory management of the SVC aneurysm in this patient stands in contrast with the majority of the cases found in the literature. Most patients were treated prophylactically for theoretical complication.

## 3. Discussion

SVC aneurysms are very rare occurrences and are most often incidentally discovered on imaging. There are two main categories of SVC aneurysms described within the literature: fusiform aneurysms first described in 1949 by Abbott [2, 6] and saccular aneurysms first reported by Lawrence and Burford in 1956 [7]. Investigation of SVC aneurysms entails the utilization of imaging modalities such as computed tomography, magnetic resonance imaging, and venography.

The etiology of SVC aneurysms is not well known. It is believed that most SVC aneurysms are congenital without abnormality identified on histopathology [8]. There are reports of a pathological deficiency in the longitudinal muscle layer within the wall of the SVC, as well as an association with cystic hygromas [8]. A study conducted by Joseph et al. indicated that five of 15 patients who were found to have a cystic hygroma had an aneurysm of the superior vena cava [9]. This association may be related to venous and lymphatic systems sharing similar embryological origins.

Treatment decisions are influenced by the size and type of SVC aneurysm. In the case of a fusiform SVC aneurysm, the general consensus is that conservative management is recommended along with observation. Developing a treatment plan for an individual with a saccular aneurysm requires more consideration. Long-term anticoagulation with agents such as warfarin or rivaroxaban could be considered to prevent thrombus and, therefore, prevent pulmonary thromboembolism. As the aneurysm grows in size, the risk of rupture increases. Anticoagulation with the above agents in the setting of aneurysm rupture increases morbidity and mortality. Therefore, aspirin therapy has been proposed to both reduce thromboembolic events and decrease morbidity in the event of venous aneurysm rupture [4]. In cases of larger saccular SVC aneurysms, even those that are asymptomatic, prophylactic surgical resection has been recommended [5].
